# Effect of Acidic Environment and Tooth Brushing on the Color and Translucency of 3D-Printed Ceramic-Reinforced Composite Resins for Indirect Restorations and Hybrid Prostheses

**DOI:** 10.3390/polym17202772

**Published:** 2025-10-16

**Authors:** Sarah M. Alnafaiy, Nawaf Labban, Alhanoof Saleh Aldegheishem, Saleh Alhijji, Refal Saad Albaijan, Saad Saleh AlResayes, Rafa Abdulrahman Alsultan, Abeer Mohammed Alrossais, Rahaf Farhan Alanazi

**Affiliations:** 1 Department of Clinical Dental Sciences, College of Dentistry, Princess Nourah bint Abdulrahman University, P.O. Box 84428, Riyadh 11671, Saudi Arabia; asaldegheishem@pnu.edu.sa; 2 Department of Prosthetic Dental Sciences, College of Dentistry, King Saud University, P.O. Box 60169, Riyadh 11545, Saudi Arabia; nalabban@ksu.edu.sa (N.L.); salresayes@ksu.edu.sa (S.S.A.); 3 Department of Dental Health, College of Applied Medical Sciences, King Saud University, P.O. Box 10219, Riyadh 12372, Saudi Arabia; smalhijji@ksu.edu.sa; 4 Department of Prosthodontics, College of Dentistry, Prince Sattam bin Abdulaziz University, P.O. Box 173, Al-Kharj 11942, Saudi Arabia; r.albaijan@psau.edu.sa; 5 College of Dentistry, Princess Nourah bint Abdulrahman University, P.O. Box 84428, Riyadh 11671, Saudi Arabia; alsultanrafa2@gmail.com (R.A.A.); abeer.rossais@gmail.com (A.M.A.); rahfaalanazi@gmail.com (R.F.A.)

**Keywords:** CAD/CAM, ceramic-reinforced composites, color, hybrid dentures, nanoceramics, translucency

## Abstract

This study evaluated the effect of acidic environment and tooth brushing on the color stability and translucency of stained 3D-printed ceramic-reinforced composite (CRC) resins for indirect restorations and hybrid prostheses. Twelve specimens were prepared from each 3D-printing resin material: Ceramic Crown (CC), OnX (ONX), and Tough 2 (T2), and one CAD/CAM milling resin, Lava Ultimate (LU). After preparation, all specimens were stained, then immersed in either water or citric acid. Subsequently, the specimens underwent simulated tooth brushing for 3650 cycles. Color stability (ΔE) and translucency parameter (TP) were measured using a spectrophotometer. Data were analyzed using ANOVA, post hoc Tukey tests, and independent Student t-tests (α = 0.05). Material type, immersion medium, and their interaction did not significantly influence the mean ΔE (*p* > 0.05). The lowest ΔE value was for LU in acid (ΔE = 1.11 ± 0.39), and the highest for T2 in water (ΔE = 2.09 ± 1.47). Except for ONX and LU in acid, all materials had ΔE values above the perceptibility threshold (ΔE = 1.2). The mean TP was significantly affected by material type, immersion medium, and their interaction (*p* < 0.05). The lowest TP value was for group CC in acid (0.91 ± 0.26); the highest was for group LU in acid (6.24 ± 0.56). After immersion and subsequent tooth brushing, TP values decreased for all materials. Exposure to an acidic environment and tooth brushing did not affect color stability but significantly reduced translucency. Both the 3D-printed CRCs and milled resin material displayed comparable color stability below clinically acceptable thresholds, though the translucency of 3D-printed materials remained lower compared to milled material.

## 1. Introduction

Computer-aided design and computer-aided manufacturing (CAD/CAM) techniques have facilitated the widespread clinical adoption of efficient and precise methods for fabricating durable and esthetic dental restorations [1]. In that context, the popularity of ceramic-reinforced composite (CRC) resin materials for dental restorations has grown tremendously in the past 15 years. These materials combine the esthetic properties of ceramics with the adaptability and handling advantages of composite resins [2,3]. Incorporating ceramic particles into the resin matrix increases the material’s wear resistance, strength, and esthetic characteristics [4]. Initially, CRCs were intended to be used with CAD/CAM milling, and this method has received broad acceptance in clinical settings [2]. Laboratory research and clinical trials have defined their indications, limitations, and clinical applications [1,5,6,7,8,9,10,11,12,13,14,15]. However, the milling process is limited by restricted movement of cutting devices, fixed milling bur sizes, material waste, and the inability to recycle waste material [4].

Advances in dental materials science have recently enabled the development of 3D-printed CRCs for use as definitive dental restorations. The first generation of 3D-printed CRCs is now available to clinicians and dental technicians, thereby expanding the range of materials for restorative dentistry [2]. Manufacturers have stated that 3D-printed CRC is suitable for long-term indirect restorations as veneers, crowns, onlays, inlays, and artificial teeth in dental prostheses [4]. In the past few years, 3D-printed CRC has extended its range of application to include fixed implant-supported hybrid prostheses; 3D-printed CRCs provide an alternative to subtractive-manufactured CAD/CAM materials. The layer-by-layer additive manufacturing process inherent to 3D-printing not only provides enhanced design flexibility but also enables the concurrent fabrication of multiple restorations, thereby reducing both production time and costs compared to CAD/CAM milling methods [16]. Furthermore, 3D-printing enables the fabrication of complex anatomical geometries and internal structures that are difficult to achieve with milling [17]. However, the high filler content in 3D-printable resins is a significant limitation. Excess filler can impede resin flow during fabrication, increasing the risk of air bubbles and non-uniform microstructures, which compromise mechanical properties. Consequently, printable resins typically contain less filler (30–50 wt%) than those designed for subtractive manufacturing (80–85 wt%) [18,19,20]. In response to these limitations, researchers have recently focused on reinforcing polymer matrices with nanoparticles, which offer excellent corrosion resistance and durability. Furthermore, optimizing nanoparticle size, shape, and composition can further enhance the material’s properties [18,21].

The optical properties of restorative materials are essential for replicating the appearance of natural teeth [22]. The color of a material is typically characterized using the Munsell color system. This system defines color in terms of three primary attributes: hue, lightness, and chroma [23]. In addition to color, translucency is a critical factor affecting the esthetics of dental restorations. It refers to the ability of a material to permit the visibility of an underlying background. The degree of translucency in dental materials results from the interaction of incident light with reflection, absorption, scattering, and transmission processes [14,24,25,26]. Numerous studies have investigated the color and translucency of conventional and milled dental CRCs [4,15,23,27,28,29,30,31]. However, there is limited information regarding the color and translucency properties of 3D-printable dental materials. These limited studies have concluded that 3D-printed CRCs do not yet match conventional or milled resins in optical properties [32,33,34,35,36,37].

The longevity of dental restorations is affected by mechanical and chemical influences [38]. Mechanical influences include excessive occlusal force from malocclusion and parafunctional activities such as bruxism and clenching. Chemical influences include exposure to acids from extrinsic sources, such as carbonated drinks, fruit juices, sports drinks, acidic foods, and certain medications. Intrinsic sources include elevated gastric acid due to medical conditions like gastroesophageal reflux disease (GERD) and bulimia nervosa [38]. Normally, saliva keeps the oral cavity at an average pH of 6.7–7.2. Acidic exposure can lower the pH below 5.52. At this level, significant damage to both hard and soft oral tissues can occur. Acidic conditions can also alter the optical and physical characteristics of dental restorative materials and tooth structures, leading to surface degradation and increased surface roughness [39,40]. Selection of optimal esthetic restorative materials requires an understanding of the behavior of ceramic and polymeric materials under exposure to an acidic environment. The previous literature demonstrates that acidic substances alter the color and translucency and surface properties of various restorative materials over time [38,39,40]. Nevertheless, a recent review [41] indicated that the evaluation and characterization of 3D-printing CRCs for dental restorations with respect to esthetic properties remains insufficient.

There is a significant gap in the literature which underscores the need for comprehensive laboratory and clinical studies on current 3D-printed CRC materials, especially materials intended for stress-bearing dental applications such as dental crowns and bridges and fixed implant-supported hybrid prostheses. In recent years, there has been a marked increase in patient preference for hybrid dentures [35]. Consequently, evaluating the performance of emerging hybrid denture materials is critical, with particular attention to influences on optical properties resulting from environmental exposure or prolonged use.

The aim of this study is to evaluate the effect of an acidic environment and simulated tooth brushing on the color stability and translucency of stained 3D-printed CRCs resins intended for indirect restorations and hybrid dentures, and to compare the outcome with a milled CRC resin. The first hypothesis is that the acidic environment would not have a significant effect on the color stability and translucency of the tested 3D-printed CRCs. The second hypothesis is that the color stability and translucency of the tested 3D-printed CRCs are comparable with milled CRC after exposure to acidic environment.

## 2. Materials and Methods

### 2.1. Materials

This study evaluated three new 3D-printing resin materials—Ceramic Crown (CC) [42], Tough 2 (T2) [43], and ONX (ONX) [44]—all sourced from the same manufacturer, and one CAD/CAM milling material, Lava Ultimate (LU). Table 1 provides detailed information about these materials.

### 2.2. Specimen Preparation

**Figure 1:**
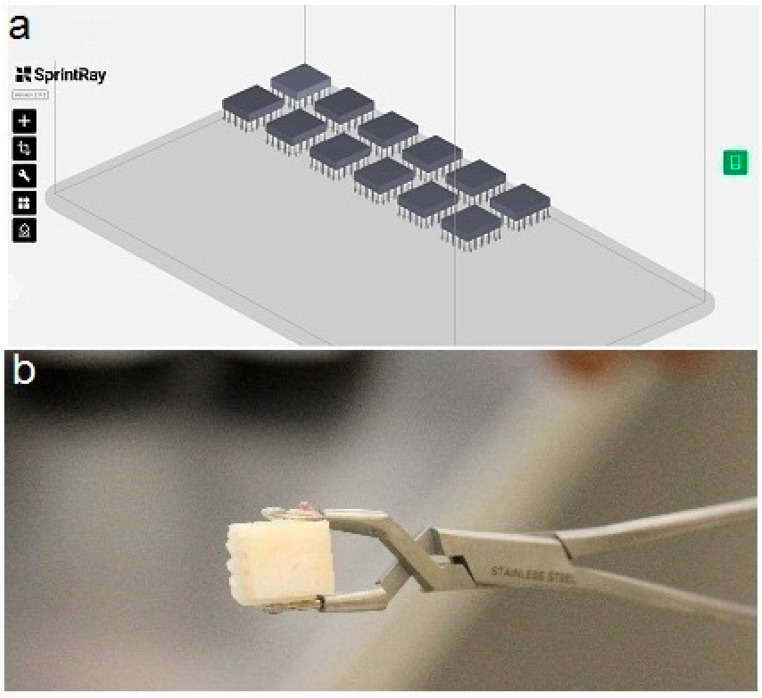
(**a**) CAD design of the specimen, (**b**) 3D-printed specimen.

A priori sample size calculation with 0.35 effect size [12], 0.80 statistical power, and an alpha error of 5% required a minimum of 12 specimens per group to demonstrate a statistically significant difference. Accordingly, 12 rectangular specimens measuring 12 × 14 × 2.5 mm^3^ were fabricated from each material. LU material specimens were sectioned under water coolant from 12 × 14 × 18 mm^3^ CAD/CAM blocks using an automated saw (IsoMet 1000 Precision, Buehler, Bluff, IL, USA). For the 3D-printing resin materials, specimens were digitally designed in CAD software (v.2.8.3 RayWare, SprintRay, Los Angeles, CA, USA) (Figure 1a) to the specified dimensions and saved as stereolithography (STL) files. The STL files were imported into the printing software, and specimens were printed using a 3D-printer (Pro S, SprintRay, Los Angeles, CA, USA) at a printing orientation of 0° and a layer thickness of 50 µm for CC and ONX and 100 µm for T2. The printed specimens (Figure 1b) were washed and dried according to the manufacturer’s instructions (ProWash S, SprintRay, Los Angeles, CA, USA). Post-curing was performed for all specimens (ProCure 2, SprintRay, Los Angeles, CA, USA) using the manufacturer-recommended curing times programmed into the device for each material.

### 2.3. Specimen Staining

**Figure 2:**
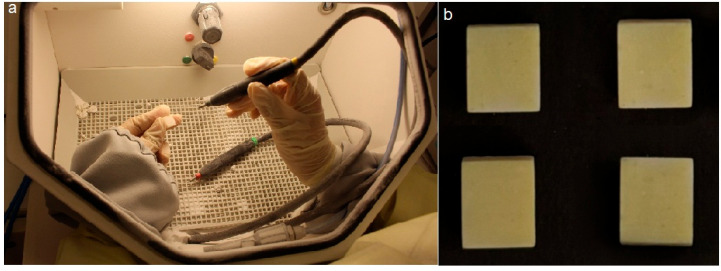
(**a**) Air-particle abrasion procedure of the specimen. (**b**) Stained specimens.

For the specimen staining, the OptiGlaze kit (GC International AG, Tokyo, Japan) was used with all the materials according to the manufacturer’s instructions. The specimens were first air-particle-abraded with 100 μm alumina particles at 2 bar PSI, cleaned using steam water, and dried (Figure 2a). A thin layer of Ceramic Primer II (GC International AG, Luzern, Switzerland) was applied to the specimens, followed by application of OptiGlaze yellow stain in thin layers with a brush [12]. The specimens were light-cured (Sprintray, Los Angeles, CA, USA) for 40 s, and the specimens (Figure 2b) were allocated to two groups.

### 2.4. Surface Treatment of the Specimens

Specimens from each material were randomly and equally assigned to either distilled water (*n* = 6) or 0.3% citric acid (pH 3.2) (*n* = 6). All specimens were incubated at 23 °C for 7 days in their respective storage media. The pH of the citric acid was monitored regularly, and the solutions were replaced daily. After the immersion period, each specimen was removed and washed thoroughly for 10 min. Subsequently, the specimens underwent simulated brushing using a tooth brushing machine (V-8, Omron, Kyoto, Japan) equipped with a toothbrush (Oral-B, P40, Procter and Gamble, Cincinnati, OH, USA). Each specimen was fixed in the specimen container, which was filled with a slurry of toothpaste and distilled water in a 1:1 ratio. The brushing parameters included a load of 2N and a bristle travel length of 10 mm at a speed of 5 mm∙s^−1^ [17]. The specimens were subjected to 3650 brushing cycles, equivalent to 7300 tooth brushing strokes, simulating one year of clinical use [9].

### 2.5. Measurement of Color and Translucency

A spectrophotometer (Labscan XE; Hunter Associates Laboratory, Reston, VA, USA) was used to analyze the color of specimens at baseline (T0) and after immersion and simulated tooth brushing (T1). The device features an integrated sphere with a D65 illumination curve and a 10° observation angle, and was operated within a wavelength range of 360 to 740 nm. L*, a*, and b* color values for each specimen were measured against both black and white backgrounds [45]. The color difference (ΔE) of the specimen was measured following the ISO Technical Specification (ISO/TR 28642:2016) [46] using the CIELab Equation (1).

(1) $$\Delta E={\left[{\left(∆L^{*}\right)}^{2}+{\left(∆a^{*}\right)}^{2}+{\left({∆b}^{*}\right)}^{2}\right]}^{1/2}$$

The differences in L*, a*, and b* values before and after immersion are denoted as ΔL*, Δa*, and Δb*. Based on previous reports [47,48], the perceptibility threshold (PT) and acceptability threshold (AT) in dentistry are ∆E = 1.2 and 2.7, respectively. These thresholds were applied in this study.

Translucency was quantified by applying the translucency parameter (TP) equation to the L*, a*, and b* color measurements obtained over both white and black backgrounds. (2) [49,50]:

(2) $$\mathrm{TP}={\left[{\left(L_{w}^{*}-L_{b}^{*}\right)}^{2}+{\left(a_{w}^{*}-a_{b}^{*}\right)}^{2}+{\left(b_{w}^{*}-b_{b}^{*}\right)}^{2}\right]}^{1/2}$$

L* indicates the difference between light and dark colors. The a* value measures the red–green chromatic scale, and b* measures the yellow–blue chromatic scale [17]. Subscripts w and b represent color coordinates measured over white and black backgrounds, respectively.

### 2.6. Scanning Electron Microscopy (SEM) Evaluation of the Specimens

A scanning electron microscope (SEM; JSM-6610LV, JEOL, Tokyo, Japan) qualitatively assessed specimens before and after immersion in storage media. Gold sputter-coating was applied to the specimens, and images were taken at 1000× magnification, with a 10 µm working distance and an accelerating voltage of 10 kV.

### 2.7. Data Analysis

All data analysis was performed using SPSS v.22 software (IBM SPSS, Chicago, IL, USA). The ∆E and TP data were expressed as means and SDs for all groups to present the descriptive analysis. The Shapiro–Wilk test revealed normal distribution of ∆E and TP values, and hence parametric tests were used (*p* > 0.05). For inferential statistics, ∆E and TP data were analyzed to observe any potential interactions between the materials and immersion medium using a two-way ANOVA test. Mean ∆E and TP values were compared between materials for each immersion medium using one-way ANOVA, and between immersion media for each material using an independent Student t test. Post hoc Tukey tests assessed multiple TP comparisons between materials. Statistical significance was set at *p* < 0.05.

## 3. Results

### 3.1. Color

The mean ΔE was not significantly affected by material type, immersion media, or their interaction (Table 2) (*p* > 0.05; two-way ANOVA).

**Figure 3:**
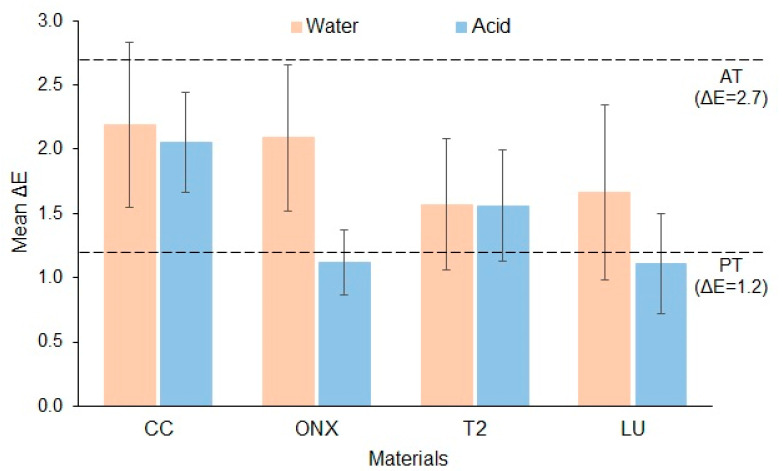
Mean color change (ΔE) of the stained materials (perceptibility threshold, PT; acceptability threshold, AT).

Figure 3 presents the mean ΔE values with standard deviation bars for each material. The lowest and highest ΔE values were observed for LU in acid (ΔE = 1.11 ± 0.39) and CC in water (ΔE = 2.19 ± 0.64), respectively. With the exception of ONX and LU in acid, all materials exhibited ΔE values above the PT (ΔE = 1.2). The comparison of ΔE values across materials and immersion media is presented in Table 3. The results indicated no significant differences in ΔE between the materials or storage media (*p* > 0.05).

### 3.2. Translucency Parameter (TP)

The mean TP was significantly influenced by the type of material, the immersion media, and their interaction (Table 4) (*p* < 0.05; two-way ANOVA).

**Figure 4:**
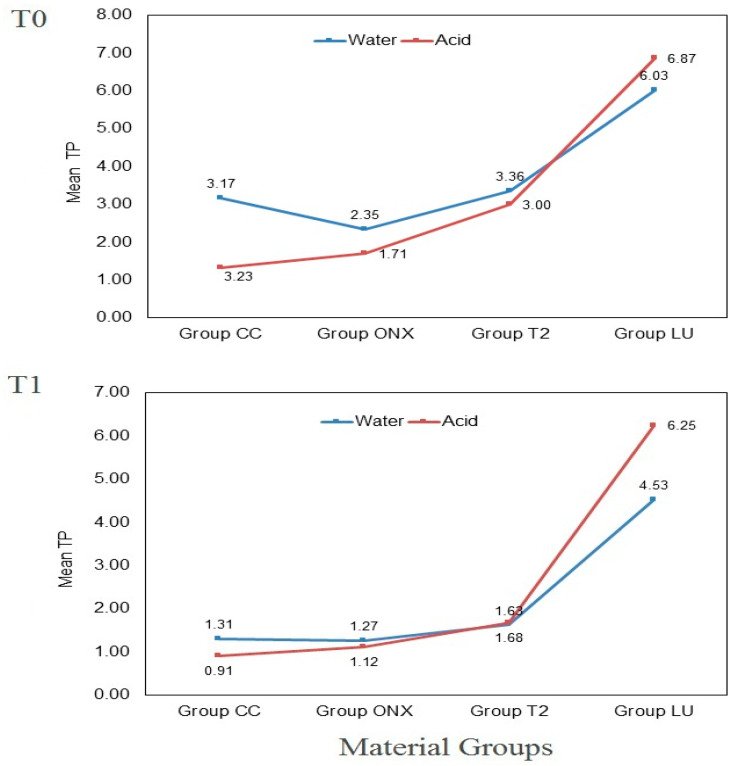
Mean TP of the specimens at different measurement points.

Figure 4 presents the mean TP for the tested materials before (T0) and after (T1) in water and acid immersion media. Before immersion (T0), group LU (6.87–6.03) and group ONX (1.71–2.35) demonstrated the highest and lowest TP, respectively. After immersion in the respective media (T1), groups ONX (1.27 ± 0.68) and LU (4.53 ± 0.79) showed the lowest and highest TP values among the water immersion specimens. For the acid immersion specimens, group CC (0.91 ± 0.26) and group LU (6.24 ± 0.56) demonstrated the lowest and highest TP values, respectively.

Table 5 presents the comparison of TP values between the material groups for the two immersion media at different measurement points. At T0, significant differences in TP values were observed independently for water and acid immersion specimens (*p* < 0.001). However, the specimens did not show any significant differences in TP between water and acid immersion specimens, implying that the TP was similar at baseline, before immersion in the medium. Similarly, at T1, TP values were observed independently for water and acid immersion specimens (*p* < 0.001). The comparison between the water and acid groups showed a non-significant difference, except for group LU (*p* = 0.002).

The comparison of mean TP of the materials between measurement points for water and acid immersion media is presented in Table 6. All materials exhibited a decline in TP values from T0 to T1, regardless of the immersion media. For the water media, groups CC, T2, and LU showed a significant decline in TP values from T0 to T1 (*p* < 0.001). The immersion of materials in acid media showed a significant decline in TP for materials CC and T2 (*p* < 0.01).

### 3.3. SEM Analysis

**Figure 5:**
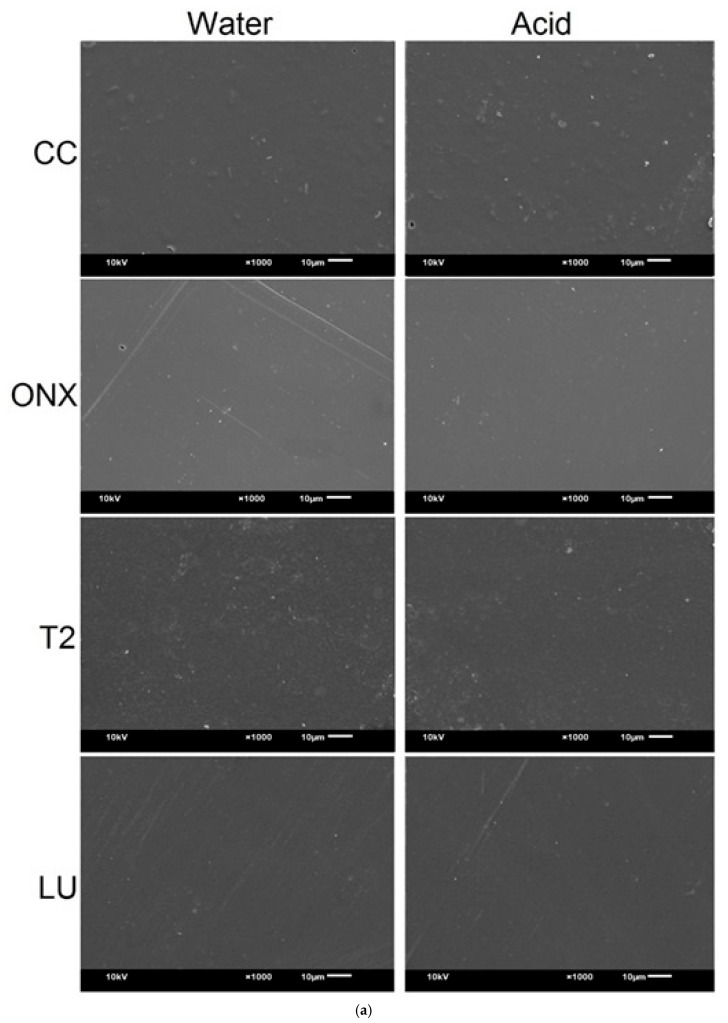

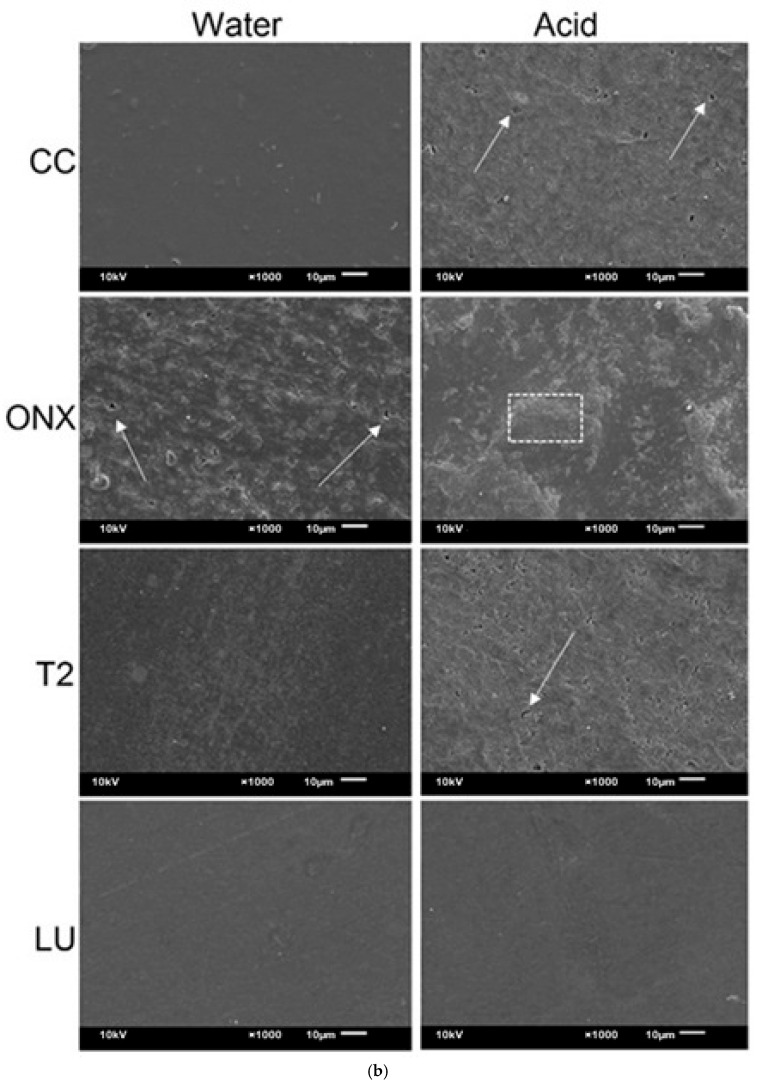
(**a**) Scanning electron microscope image (1000× magnification) of the materials at baseline. (**b**) Scanning electron microscopy images of the stained specimens after immersion and tooth brushing (1000×). The arrows indicate pits and irregular flecks. The rectangular box denotes craters and erosions of the ONX specimen stored in acid.

Figure 5a,b presents the SEM images of the study materials before and after immersion in the respective media. Prior to immersion, CC and T2 displayed slightly scattered filler particles, whereas ONX exhibited a more homogeneous surface, followed by LU. Following immersion, however, a notable shift occurred: all materials except LU showed substantial surface changes. Specifically, ONX, when immersed in both water and acid, demonstrated pronounced surface alterations, including increased roughness, irregular flecks, and erosive craters (Figure 5b), in comparison to the baseline and other materials. Group T2 material in acid exhibited a roughened surface and irregular flecks compared to the water medium, and group CC in acid demonstrated a similar surface topography to T2. In contrast, CC in water and LU in both water and acid demonstrated a homogeneous surface similar to their images before immersion. Overall, ONX and T2 materials exhibited significant changes in their surface topography compared to their surfaces prior to immersion.

## 4. Discussion

The drive for durable and visually appealing treatments has advanced new materials and methods, especially for indirect restorations [1]. The main objective of any dental restorative materials is to accurately replicate the properties and visual characteristics of natural dental tissues [2]. Therefore, the selection of dental materials must consider the vulnerability of esthetic restorative materials to alterations in color and translucency, as these properties are critical factors that affect both the visual outcome and patient satisfaction [8]. The mechanical properties of the 3D-printed CRC materials tested in the current study have been analyzed recently in a previous publication [4]. However, information regarding their optical properties is lacking. Therefore, the outcome of this study could add to the existing literature to give thorough knowledge to clinicians before their use in routine clinical practice. The data analysis of the current study revealed that the acidic environment and simulated brushing did not affect ΔE, but they significantly affected the TP of the tested materials. Thus, the first hypothesis—that the acidic environment would not significantly affect the ΔE or TP of the tested 3D-printed CRCs—was partially rejected. The results of this study also indicated that 3D-printed CRCs exhibited ΔE comparable to milled CRCs. However, the TP of 3D-printed CRCs was significantly lower than milled CRCs. Based on these findings, the second hypothesis was also partially rejected.

The materials immersed in acid exhibited better color stability than those immersed in water. This difference could be related to the water absorption process, which is a significant factor contributing to color change susceptibility. Specifically, water absorption leads to expansion and plasticization of the organic matrix, thus reducing the durability of the composite resin and promoting color change [12]. Conversely, the effect of citric acid differs. Citric acid modifies ceramic particle surfaces, changing their refractive index and reducing light reflection at the ceramic–matrix interface [31]. Among the water immersion groups, group T2 followed by group LU demonstrated high color stability, whereas in the acid immersion, groups LU and ONX demonstrated high color stability and were also more resistant to acid exposure. The difference in ΔE between milled and 3D-printed CRCs was non-significant and is in disagreement with previous studies that have demonstrated superior ΔE of milled resins over 3D-printed resins [11,13,14,23]. Studies indicate that increased water sorption in 3D-printed CRCs, compared to pressed and milled CRCs, contributes to low color stability [11,23]. The increased water sorption and hydrolysis, which occur due to the slow polymerization rate that persists even after post-curing, result in low color stability [11,23]. As a result, higher residual monomers—resulting from hydrolysis and water sorption—lead to surface softening and compromised surface integrity.

Another important aspect determining the color stability of CRCs is the filler characteristics, including size, shape, and composition. The fillers are added to improve the mechanical properties, but they also influence the material’s refractive indices, thereby affecting the optical properties [12]. The a* and b* values exhibit significant differences between materials containing spherical fillers and those with irregular fillers. Specifically, for materials with irregular fillers, an increase in filler quantity leads to a decrease in the a* value and an increase in the b* value. In contrast, for materials with spherical fillers, increasing the filler quantity results in higher a* values and lower b* values. Furthermore, regardless of the type of filler, the smallest filler size consistently produces the highest light transmittance across all filler contents, whereas larger filler sizes are associated with reduced transmittance [35]. To determine the properties of 3D-printed CRCs, it is important to consider the shape, size, quantity, and distribution of fillers. The monomer content and the filler characteristics in the resin can affect its optical properties [2]. Studies show that, unlike CAD/CAM milled CRCs, 3D-printed resins need at least 50 wt% filler to achieve better clinical results [18,19,20]. In this study, the 3D-printed products are new to the market, and manufacturers still control the manufacturing process without revealing the complete constituents of the resins. However, a previous study has shown that the polymer matrix of CC and ONX is likely to be a methacrylate, and the filler is spherical particles of SiO_2_ (0.2–0.5 μm) and irregular particles of SiO_2_ and Yb_2_O_3_ (1–5 μm), making up to 50% and 38% for CC and ONX, respectively. In contrast, the polymer matrix of T2 is made of urethane dimethacrylate, and the filler is up to 34% spherical particles of SiO_2_ (0.2–1.5-μm). These data confirm that the ΔE of the tested 3D-printed CRCs is material dependent.

Evaluating clinical and research color outcomes in real-life contexts requires comparison with PT and AT, regardless of statistical analysis. Dental and restorative discoloration is clinically significant when it reaches the human visual PT or AT, benchmarks for defining color differences [48]. However, the gold standard for color threshold values is still controversial, and therefore AT and PT values have differed between studies. In this study, the threshold values were selected based on the most recent description [47]. The ΔE of the tested materials in the current study was below the AT (ΔE < 2.7). Specifically, the ΔE of all materials immersed in water was above the PT (ΔE > 1.2) but below the AT. For materials immersed in acid, the ΔE of group CC and T2 was above the PT but below the AT, while the ΔE of group ONX and LU was below the PT. Previous studies [13,14,23] evaluating ΔE of 3D-printed resins have reported that the materials in their study demonstrated ΔE values above the AT, contrary to the findings of this study. One possible reason for the low ΔE in the current study could be attributed to the specimen staining.

In the current study, the tested materials underwent staining as opposed to polishing per the manufacturer’s instructions [3]. Whereas polishing removes a superficial layer to enhance surface smoothness, staining applies a thin coating of material to replicate the natural color, translucency, and features of the patient’s existing teeth. The presence of stained layers affects surface roughness, mechanical resistance, and esthetic outcomes across various restorative materials compared to the polishing process [3].

Translucency is vital in restorative dentistry for replicating the optical qualities of natural teeth, which vary by thickness and anatomical site. Highly translucent materials are especially important in CAD/CAM technologies, allowing dental restorations to better mimic natural teeth [1]. In this study, the TP values at T0 ranged from 1.71 to 6.87 across all groups, with 3D-printed ONX exhibiting the lowest and CAD/CAM milled LU the highest TP values. At T1, specimens immersed in acid displayed TP values between 0.91 and 6.25 (LU > T2 > ONX > CC), while those immersed in water ranged from 1.27 to 6.25 (LU > T2 > CC > ONX). Notably, all materials demonstrated a decrease in TP value from T0 to T1. However, the decrease was significant for groups CC, T2, and LU in water media and groups CC and T2 in acid media. This confirmed that an acidic environment followed by tooth brushing affected the TP values of two of the tested 3D-printed CRCs. This decrease in TP aligns with findings from previous studies [12], which have indicated that surface texture is modified by both environmental changes and toothbrush abrasion in the oral cavity. Specifically, a difference in refractive index between ceramic and resin fillers produces a roughened and porous surface, which alters light refraction and reduces both translucency and perceived color.

In this study, the TP differed between the 3D-printed and milled groups. Furthermore, within the 3D-printed materials, TP values varied depending on whether they were immersed in water or acid media. LU exhibited increased TP among the materials and was also resistant to change in TP after exposure to an acidic environment. This is likely due to the incorporation of 80% silica and zirconia nanoparticles within a strongly cross-linked TEGDMA resin matrix. The nanometer-sized filler particles have diameters smaller than the wavelength of visible light. As a result, these particles reduce light scattering and enhance light transmission [5]. Dental restorative materials are expected to exhibit a translucency parameter (TP) value similar to that of enamel, which ranges from 15 to 19 for a 1 mm specimen [1]. For reference, the TP value for a 1 mm thick LU specimen has been reported as 17.93 [15]. In a previous study, both 1 mm thick specimens of LU and 3D-printed permanent crown resin showed no significant difference in TP values, with measurements of 12.93 and 12.52, respectively [1]. Another study assessing the TP of five different 1 mm thick 3D-printing materials for printing crowns and bridges under various storage conditions found only minor changes in TP values [23]. These findings contrast with the results of the current study, where all tested 3D-printed CRCs exhibited significantly lower TP values compared to milled LU.

The specimen thickness utilized in this study differed from that reported in previous research. Typically, dental crowns and bridges measure 1 to 2 mm in thickness, whereas materials used for implant-supported temporary prosthesis abutments are generally manufactured with greater thickness [23]. For consistency, we prepared specimens at a 2.5 mm thickness. The thickness of a restoration can greatly affect its translucency; as thickness increases, TP values decrease. Several factors influence the thickness of restorative materials, including the type of tooth being restored, the technique used, the amount of coverage needed, and the material’s physical and mechanical characteristics [27]. A decrease in material thickness results in reduced absorption and increased light transmission. Thickness, combined with the material’s absorption and scattering properties, determines the proportions of reflected, absorbed, and transmitted light [29]. However, the findings of the current study may not extend to thinner specimens and must be interpreted with this limitation.

This study has several limitations. A major limitation is the restricted access to detailed information about 3D-printed resin constituents due to intellectual property rights. This restriction, especially regarding the filler characteristics and the resin matrix, prevents a thorough assessment and understanding of the materials in this study. Another notable limitation is the exclusive use of a 0° build orientation and 50 (CC and ONX) and 100 µm (T2) layer thickness for material printing, which restricts the generalizability of the findings. Currently, standardized guidelines for optimal build orientation and layer thickness in 3D-printed CRCs are lacking. This leads to inconsistent processing protocols, contributing to variability in mechanical and esthetic performance and potentially compromising the durability of 3D-printed restorations. Evaluating a broader range of printing angles and layer thicknesses would provide a more thorough assessment of the optical properties of the materials examined. The experimental protocol included immersion, simulated brushing, and a controlled laboratory setting with uniform thickness, a single pH value, and consistent specimen shade. However, these laboratory conditions do not accurately reflect the intricacies of the clinical oral environment, which involves varying salivary flow, fluctuating oral pH, and the interplay of mechanical and chemical hygiene practices. These intra-oral factors, which were excluded from this study, may markedly influence the optical properties of the materials. Additionally, the study was limited to three 3D-printed materials from one manufacturer and a single milled control, which may restrict the generalizability of the findings to other material types and manufacturers. Future studies should focus on evaluating different printing orientations, specimen thicknesses, and different shades of the tested material. Furthermore, other surface, mechanical, and biocompatibility properties of the tested materials need evaluation. In addition, it would also be interesting to rank the materials and their alternatives based on the mechanical, thermal, and tribological performance criteria using the R-method that could aid in decision making [51].

## 5. Conclusions

Within the limitations of this current study, it was concluded that exposure to an acidic environment and simulated tooth brushing did not affect color stability but significantly reduced translucency. Both the 3D-printed CRCs and milled resin material displayed comparable color stability below clinically acceptable thresholds, though the translucency of 3D-printed materials remained lower than that of milled resin material.

## Figures and Tables

**Table 1:** Details of the materials used in the study.

Material/Type /Application	Shade/Abbreviation	Manufacturer	Composition	Batch Number
Ceramic Crown/3D-Printing resin/Indirect restorations	A1/CC	SprintRay, Los Angeles, CA, USA	* Methacrylic acid esters, pigments, photoinitiators. 50% irregular and some rounded silica and ytterbium oxide (1.0–5.0 μm) [2,42]	SRI 0202057
OnX/3D-Printing resin/Hybrid dentures	A1/ONX	SprintRay, Los Angeles, CA, USA	* Methacrylate monomers and oligomers, acrylic monomers, photoinitiators [44]	SRI 0202057
ONX Tough 2/3D-Printing resin/Hybrid dentures	A1/T2	SprintRay, Los Angeles, CA, USA	* Methacrylic acid esters, pigments, photoinitiators [43]	SRI 0202164
Lava Ultimate/Milling/Indirect restorations	A1/(LT)/LU	3M ESPE, St. Paul, MN, USA	20 wt% cross-linked polymer matrix (TEGDMA) and up to 80 wt% cluster nanofillers containing 4–11 nm zirconia, 20 nm silica, and aggregated zirconia or silica	5074A1-LT

* The actual composition of the products is not revealed by the manufacturers. TEGDMA—triethylene glycol dimethacrylate.

**Table 2:** The effect of the material and immersion media on ΔE.

Source	Type III SS	df	Mean Square	F	*p*-Value
Corrected model	7.371	7	1.053	1.86	0.10
Intercept	134.268	1	134.268	237.149	<0.001 *
Immersion media	2.034	1	2.034	3.592	0.07
Materials	3.586	3	1.195	2.111	0.11
Immersion media × materials	1.752	3	0.584	1.031	0.39
Error	22.647	40	0.566		
Total	164.287	48			
Corrected total	30.018	47			

* Statistically significant (two-way ANOVA).

**Table 3:** Comparison of mean ΔE between materials and immersion media.

Parameter	Immersion Media	Materials				*p*-Value ^†^
		CC	ONX	T2	LU	
ΔE	Water	2.19 ± 0.64	2.09 ± 0.57	1.57 ± 0.51	1.66 ± 0.68	0.63
	Acid	2.05 ± 0.39	1.12 ± 0.25	1.56 ± 0.43	1.11 ± 0.39	0.06
*p*-value ^‡^		0.66	0.14	0.99	0.12	

**^†^** One-way ANOVA; ^‡^ independent Student *t* test.

**Table 4:** The effect of the materials and immersion media on TP.

Source	Type III SS	df	Mean Square	F	*p*-Value
Corrected model	160.478	7	22.925	96.154	<0.001 *
Intercept	262.127	1	262.127	1099.42	<0.001 *
Immersion media	1.101	1	1.101	4.618	0.04 *
Materials	151.1	3	50.367	211.249	<0.001 *
Immersion media × materials	8.277	3	2.759	11.572	<0.001 *
Error	9.537	40	0.238		
Total	432.142	48			
Corrected total	170.015	47			

* Statistically significant (two-way ANOVA).

**Table 5:** Comparison of mean TP between materials and immersion media at different measurement points.

Materials	T0			T1		
	Water	Acid	*p*-Value	Water	Acid	*p*-Value ^†^
CC	3.17 ± 1.10	3.32 ± 0.19	0.67	1.31 ± 0.47	0.91 ± 0.26	0.10
ONX	2.34 ± 0.48	1.71 ± 0.43	0.06	1.26 ± 0.68	1.12 ± 0.25	0.63
T2	3.35 ± 1.51	2.99 ± 0.34	0.58	1.63 ± 0.13	1.67 ± 0.34	0.77
LU	6.02 ± 0.31	6.87 ± 0.92	0.06	4.53 ± 0.79	6.24 ± 0.56	0.002 *
**^‡^***p*-value	<0.001 *	<0.001 *		<0.001 *	<0.001 *	

* Statistically significant (*p* < 0.001); ^†^ one-way ANOVA, ^‡^ independent Student *t* test.

**Table 6:** Comparison of mean TP of the materials at different measurement points for water and acid immersion media.

Materials	Water (T0–T1)			Acid (T0–T1)		
	T0	T1	*p*-Value	T0	T1	*p*-Value
CC	3.17 ± 1.10	1.31 ± 0.47	*p* < 0.01 *	3.32 ± 0.19	0.91 ± 0.26	*p* < 0.01 *
ONX	2.34 ± 0.48	1.26 ± 0.68	0.30	1.71 ± 0.43	1.12 ± 0.25	0.38
T2	3.35 ± 1.51	1.63 ± 0.13	0.013 *	2.99 ± 0.34	1.67 ± 0.34	*p* < 0.01 *
LU	6.02 ± 0.31	4.53 ± 0.79	0.04 *	6.87 ± 0.92	6.24 ± 0.56	0.31

* Statistically significant (*p* < 0.001; one-way ANOVA).

## Data Availability

All the data are present in the article.
